# Basal keratin expression in breast cancer by quantification of mRNA and by immunohistochemistry

**DOI:** 10.1186/1756-9966-29-39

**Published:** 2010-04-28

**Authors:** Radzislaw Kordek, Piotr Potemski, Renata Kusinska, Elzbieta Pluciennik, Andrzej Bednarek

**Affiliations:** 1 Department of Pathology, Medical University of Lodz, 4 Paderewski St., Lodz, Poland; 2 Departments of Chemotherapy, Medical University of Lodz, 4 Paderewski St., Lodz, Poland; 3 Department of Molecular Cancerogenesis, Medical University of Lodz, 6/8 Mazowiecka St., Lodz, Poland

## Abstract

Definitions of basal-like breast cancer phenotype vary, and microarray-based expression profiling analysis remains the gold standard for the identification of these tumors. Immunohistochemical identification of basal-like carcinomas is hindered with a fact, that on microarray level not all of them express basal-type cytokeratin 5/6, 14 and 17. We compared expression of cytokeratin 5, 14 and 17 in 115 patients with operable breast cancer estimated by real-time RT-PCR and immunohistochemistry.

Despite the method of dichotomization and statistical analysis, there were cases with discordant results comparing immunohistochemistry and RT-PCR analysis. For dichotomisation based on quartiles and ROC, 14% of cases were negative on immunohistochemical examination for CK5/6, but presented high CK5 mRNA levels. There were also 48-55% cases, which were CK5/6-immunopositive, but were negative by mRNA examination. Similar discordances were observed for CK14 and CK17.

Basal keratin mRNAs did not correlate with ER mRNA levels, while immunohistochemistry produced significant relationship with ER status.

Our observation suggest that both method may produce different results in a small proportion of cases. Discordance between immunohistochemistry and RT-PCR may confound attempts to establish a simple methods for identification of basal-like tumors.

## Introduction

Heterogeneity of breast cancer at the molecular level was supported by data from cDNA microarrays [1,2]. Tumors lacking ER form three groups: a basal-like subtype, HER2-positive subtype, and a normal breast-like subtype. Basal-like subtype is characterized by multigenetic signature, usually with high expression of high molecular weight cytokeratins normally expressed in basal myoepithelial cells: keratin 5 (CK5), 14 (CK14) and keratin 17 (CK17) [1,2]. They usually express vimentin and p-cadherin, and more than 60% of them also express epidermal growth factor receptor (EGFR) [3,4].

A great interest in basal like-cancers produced attempts to determine basal-like tumors by the use of a much more easier technique such as immunohistochemistry. Unfortunately, both methods -- oligonucleotide microarrays and immunohistochemistry - do not produce identical results. In the study by Nielsen and al., immunohistochemical panel for basal-like cancers was defined as lack of ER and HER2 expression and positivity for CK5/6 or EGFR [5]. Unfortunately, this panel still presented only 76% sensitivity for basal-like tumors derived from a microarray study.

Another attempt to simplify the determination of basal-like tumors was regarding them as synonymous with "triple negative tumors", regarded as lack of ER, PGR and HER2 [6]. But according to comparative studies, as much as 15-54% of basal-like tumors defined on mRNA level, still express at least one of these markers [4,5,7-9].

Quantitative real-time RT-PCR technology provides a precise assessment of even small changes in gene expression. In this aspect, real-time RT-PCR is a much more sensitive assay when compared with oligonucleotide microarray and could be considered as a referential method [10]. This raises the question whether microarray-based classification of breast tumors could be reconstructed or even improved by the use of data from the quantification of expression of selected genes assessed by real-time RT-PCR. Recently, there have been published some data supporting this thesis [11].

In a previous study, we have compared ER expression estimated by RT-PCR and by a routine immunostaining, and have validated which method might be more reliable for the molecular subtyping in relation with basal-type keratins and HER2 genes expression [12]. Both methods produced discordant results in a proportion of cases, and lack of prognostic relevance of ER-mRNA level has been demonstrated, whereas the assessment by immunostaining has been related to clinical outcome. Also expression of basal keratins and HER2 genes significantly differed between ER-positive and ER-negative tumors divided on the basis of immunostaining, but not by mRNA level. Whereas immunostaining results are specific for tumor cells, mRNA for the RT-PCR analysis could originate not only from cancer cells but also from normal breast epithelium, myoepithelial and stromal cells. Furthermore, due to post-transcriptional and post-translational mechanisms, the amount of detected mRNA not always directly reflects protein level.

On the contrary, qRT-PCR may produce similar results of ER expression as obtained by immunohistochemistry, but only with a very stringent and quality controlled systems [13]. Besides, immunohistochemistry still remains the gold standard for estimation of ER status in breast cancer.

Although, as stated by Reis-Filho and Tutt, "from a scientific perspective, microarray-based expression profiling analysis remains the gold standard for the identification of basal breast cancers", stringent analysis of profiles discloses that in basal-like cases there is low expression of basal cytokeratins in a few cases [2-4]. Similarly, in some luminal-type tumors there are cases with high expression of CK5 or CK14 [2,3]. As mRNA for basal-type cytokeratins may originate from myoepithelial cells forming normal breast tissue intermixed with cancer cell, or the number of cancer cells even presenting these cytokeratins may be to sparse -- in both situations false results may be obtained.

The aim of this retrospective study was to compare basal-cell-type cytokeratin expression estimated by real-time RT-PCR and by a routine immunostaining.

## Patients and Methods

### Tumor specimens and study patients

Specimens of primary tumors were consecutively obtained from 115 women with operable invasive ductal carcinomas not otherwise specified (NOS) at a time of routine surgery at the Oncology Department of Copernicus Memorial Hospital in Lodz, Poland, between 1998 and 2001. In all cases, surgical procedure was a radical mastectomy with axillary lymph node dissection. Serial sections of the tumor were obtained from archived paraffin embedded tissue blocks. The primary pathologic diagnosis was confirmed in H&E staining. Subsequent slides were stained for ER and HER2. For further mRNA analysis, fresh tumor specimens were frozen immediately after excision at -80°C. Patient characteristics are presented in table 1.

**Table 1:** Patient characteristics

Factor	Number of patients
Number of patients	115

Age (years)	
≤ 50	39 (33,9%)
> 05	76 (66,1%)

Tumour	
T1	33 (28,7%)
T2-4	82 (71,3%)

Nodal status	
Positive	56 (48,7%)
Negative	59 (51,3%)

Grade	
G1-2	63 (54,8%)
G3	52 (45,2%)

ER status	
Positive	60 (52,2%)
Negative	55 (47,8%)

CK5/6 status (IHC)	
Positive	42 (36,5%)
Negative	73 (63,5%)

CK14 status (IHC)*	
Positive	16 (14,0%)
Negative	98 (86,0%)

CK17 status (IHC)	
Positive	29 (25,2%)
Negative	86 (74,8%)

Adjuvant treatment	
Chemotherapy	66 (57,4%)
Hormonotherapy	82 (71,3%)
Radiotherapy	21 (18,3%)
Missing data	8 (7,0%)

* In one sample assessment was not possible due to technical reasons

### Immunohistochemistry and scoring

Paraffin embedded sections were routinely processed. Slides for immunostaining for ER (Dako), CK14 and CK17 (both Novocastra) were pretreated with citrate buffer in a microwave oven. CK5/6 antibody from Dako was applied following autoclaving with high pH buffer. Antibody dilutions were as follows: ER - 1:35, CK5/6 - 1:100, CK14 -- 1:20, CK17 - 1:40. All following procedures were done according to standard protocols with EnVision+ System HRP (Dako). ER nuclear staining scoring was done using the method described by McCarty et al. [14]. Tumors were considered as being positive for ER if Histo-score was above 100. The results of basal keratin membranous staining were classified as follows: negative - no staining seen in invasive cancer cells, positive -- weak or strong staining seen in invasive cancer cells. HER2 expression was examined with the commercially available Herceptest kit from Dako and score +3 denoted HER2-positive tumors.

### Real-time RT-PCR analysis

Tumor samples were stored at -80°C until mRNA extraction using TRIzol^® ^Reagent (Invitrogen Corporation, USA). Synthesis of cDNA was performed from 10 μg of total mRNA at a total volume of 70 μl using ImProm-II™ (Promega Corporation, USA) reverse transcriptase. Next, cDNA samples were diluted with sterile deionized water to a total volume of 140 μl. Volumes of 2 μl (corresponding to 0, 14 μg of total mRNA) were used for PCR. Real-time RT-PCR was performed using Rotor-Gene™ 3000 (Corbett Research). Sequences of primers used, annealing and detection temperatures are presented in Table 2. All primers were designed to not amplify genomic DNA (usually one is positioned on exon-exon junction). Primer pairs were blasted against human genome ref_assembly 37.1 using electronic PCR on NCBI Genome Database and showed no genomic or pseudogenes PCR products.

**Table 2:** Real-time RT-PCR primers and reaction conditions

*Gene primers (5'-3')* *Forward* *Reverse*	*Annealing temperature (^°^C)*	*Detection temperature (^°^C)*	*PCR product size (base pairs)*
**Beta-2-microglobulin (*B2M*)** TGAGTGCTGTCTCCATGTTTGA TCTGCTCCCCACCTCTAAGTTG	50	81	88

**H3 histone, family 3A (*H3F3A*)** AGGACTTTAAAAGATCTGCGCTTCCAGAG ACCAGATAGGCCTCACTTGCCTCCTGC	65	72	76

**Ribosomal phosphoprotein (*RPLP0*)** ACGGATTACACCTTCCCACTTGCTAAAAGGTC AGCCACAAAGGCAGATGGATCAGCCAAG	65	72	69

**Ribosomal protein S17 (*RPS17*)** ACCCCAATGTCAAGGAGATCAAGGTCCTG TCGGCAGCCAGCTCGTGAGTAATG	64	72	87

**Estrogen receptor 1 (*ER*)** ATCTCGGTTCCGCATGATGAATCTGC TGCTGGACAGAAATGTGTACACTCCAGA	65	72	98

**Keratin 5 (CK5)** ATCGCCACTTACCGCAAGCTGCTGGAGGG AAACACTGCTTGTGACAACAGAG	65	72	102

**Keratin 17 (*CK17*)** ATGTGAAGACGCGGCTGGAGCAGGA ACCTGACGGGTGGTCACCGGTTC	65	72	109

**Keratin 14 (*CK14*)** TTTGGCGGCTGGAGGAGGTCACA ATCGCCACCTACCGCCGCCTG	65	72	109

All reactions were made in triplicate. Detection of PCR products was performed with SYBR™ green I using qPCR Core kit for SYBR™ green I (Eurogentec, Belgium). Expression levels of target genes were normalized using four housekeeping genes: B2 M, H3F3A, RPLP0, and RPS17. Relative gene expression was calculated with the use of the mathematical model described by Pfaffl.

### Statistical analysis

Mann-Whitney U test was employed to evaluate significance of differences in mRNA level between groups. Dichotomized values of mRNA level were compared with immunohistochemistry using the matched pairs Liddell's exact test and Scott's π test. Data were analyzed with respect to sensitivity and specificity derived from the receiver operating characteristic (ROC) and immunohistochemistry was regarded as a referential test. Kendall's rank correlation (τ) was used to test the strength of an association between expression of genes. Pearson's χ^2 ^test or Fisher's exact test were used to test for contingency between dichotomized values of basal keratin expression (negative and positive) and values of other histopathological parameters. All results were considered statistically significant when two-sided p was less than 0.05.

## Results

In 73 cases (63,5%) identified immunohistochemically as being CK5/6-negative, mean CK5 gene expression was significantly lower, than in cases classified by immunostaining as being CK5/6-positive (table 3, p = 0,001). Similar results were observed for CK14 and CK17 (p = 0,007 and p < 0,001, respectively; table 3).

**Table 3:** mRNA of respective basal keratin genes depending on their status assessed by immunohistochemistry

Status by IHC	mRNA level		p value
		
	Median; range	Mean ± SD	
CK5/6 negative	24.69; 0.00-4495.16	206.67 ± 727.20	0,001
	
CK5/6 positive	192.92; 0.00-3066.48	424.48 ± 731.51	

CK14 negative	67.50; 0.00-6615.26	209.45 ± 684.34	0,007
	
CK14 positive	250.52; 0.00-10569.08	1480.20 ± 2958.21	

CK17 negative	0.15; 0.00-22.22	0.69 ± 2.47	<0,001
	
CK17 positive	1.15; 0.01-26.44	3.11 ± 5.49	

The comparisons between dichotomized values of CK5-mRNA level and CK5/6 immunohistochemical status demonstrated, that despite the method of dichotomization and statistical analysis, there were cases with discordant results comparing immunohistochemistry and RT-PCR analysis. For two methods of dichotomisation (quartiles and based on ROC; the ROC curve analysis was performed assuming that immunostaining was a reference test), there were still 48-55% cases, which were CK5/6-immunopositive, but were negative by mRNA examination. Similarly, 14% of cases were negative on immunohistochemical examination, but presented high mRNA levels. Similar discordances were observed for CK14 and CK17.

Highly significant, moderate, positive correlations between mRNA levels of CK5 and CK14 (τ = 0.40, 95%CI 0.29-0.51, p < 0,001), between CK5 and CK17 (τ = 0.51, 95%CI 0.40-0.62, p < 0,001), and between CK14 and CK17 (τ = 0.36, 95%CI 0.25-0.47, p < 0,001) were observed.

When samples were divided in respect of basal keratins status on the basis of immunohistochemistry, significant difference in ER-mRNA level between positive and negative ones was found. We also observed significant relationship between basal keratin expression and ER status, when both were estimated by immunohistochemistry. Tumours positive for these keratins usually lacked ER receptor (table 4, 5). To the contrary, basal keratin mRNAs did not correlate with ER mRNA levels. When a group of 53 cases samples positive for basal keratins on the basis of mRNA assessment was selected, there was no significant difference in mean ER-mRNA level when compared with negative ones. Similar analyses were performed assuming other cut-off points in the process of dichotomization of basal keratin mRNAs: < median vs ≥ median, Q1-3 vs Q4, and log2 ratio (<0.65 vs ≥ 0.65). There were no significant differences in ER mRNA level regardless of the cut-off point selected (p value: 0,752, 0,331, and 0,059, respectively). In the last analysis, when log2 ratio (<0.65 vs ≥ 0.65) cut-off point was selected, only 5 cases were classified as being negative for basal keratin mRNA, whereas remaining 110 cases were classified as being positive.

**Table 4:** Relations between basal keratins expression and ER status assessed by immunohistochemistry

Basal keratin	ER		p value
		
	Negative	Positive	
CK5/6			
Negative	20	53	<0,001
Positive	35	7	

CK14			
Negative	39	59	<0,001
Positive	15	1	

CK17			
Negative	30	56	<0,001
Positive	25	4	

The table contains numbers of patients

**Table 5:** Relationship between ER and basal keratin status assessed by immunohistochemistry

Basal keratin status	ER status (number of patients)		p value
		
	Negative	Positive	
CK5/6 and CK14 and CK17 negative	18	52	<0,001
	
CK5/6 or CK14 or CK17 positive	37	8	

## Discussion

Basal-like breast cancers recently have raised a great interest not only regarding clinical differences, but also in relation with new therapeutic possibilities. The vast majority of BRCA1 mutation-related breast tumors represent basal-like subtype. Moreover, Turner et al. have recently reported the high prevalence of BRCA1 downregulation in sporadic basal-like breast cancer [15]. There are some promising data that platinum-based chemotherapy may be more effective in patients with BRCA-1 germline mutations or in "triple-negative" breast cancer [16,17]. These observations may emphasize the importance of an easy and simple determination of basal-like phenotype.

A microarray analysis is a very elegant and sophisticated method, but for individual genes it is equivalent to estimation of mRNA level by the use of RT-PCR. Both methods have one important weakness -- the assessment of gene expression is based on total mRNA presented in the examined tissue, not only in cancer cells - and this weakness may produce false results in a proportion of cases. In our study, in a comparison of immunohistochemistry and RT-PCR, regardless of the method of dichotomization and statistical analysis used, there were cases with discordant results. For each cytokeratin, there were cases which were regarded as being positive by one method, and negative by the other one. Fourteen percent of cases were negative for CK5/6 as assessed by an immunohistochemical examination, but presented high CK5 mRNA levels. Similar discordances were also observed for CK14 and CK17. This observation suggests that in some cases high levels of basal keratin mRNA may originate not from cancer cells but possibly also from preexisting normal myoepithelial cells. Furthermore, due to the post-transcriptional and post-translational mechanisms, the amount of detected mRNA not always directly reflects protein level. The same applies to oligonucleotide microarrays.

Similar differences were observed in an opposite direction - some cases which were positive by immunohistochemistry were regarded as being negative by real-time RT-PCR. For CK5/6, there is a theoretical possibility that cells may express only CK6 and not CK5, but the same observation was made for CK14 and CK17. Possibly, the amount of immunopositive cancer cells in the sample was too small to give positive results by RT-PCR when mRNA levels were dichotomized.

Moreover, for both types of discordances, it may be one universal explanation: because of the heteregeneity of the tumor, tissue examined by immunohistochemistry was not exactly the same tissue which was examined by real-time RT-PCR.

We have found that basal keratin mRNA does not inversely correlate with ER mRNA level. This is an interesting observation, as in the published studies with the use of microarray technology such correlation is clear [1-3]. But when our samples were divided regarding basal keratin status on the basis of immunohistochemistry results, we observed significant relationship with ER status, estimated both by RT-PCR and by immunohistochemistry. It shows that immunohistochemistry may be a better method than RT-PCR in rendering a biological difference of basal-like tumors.

Studies that were conducted to establish which immunohistochemical markers were helpful for the best definition of basal-like tumors gave different results [18-22]. Rakha et al. suggested that only expression of basal-type cytokeratins (CK5/6 and CK14) should be included in such definition [21]. In their study, no other marker was related with worse prognosis. More recently, some authors have claimed that EGFR expression should be added to the panel, and even in the absence of basal-cytokeratins, ER- and HER2-negative tumors presenting EGFR should be regarded as basal-type ones [5,20,21]. Nielsen at al. determined that 13 of 21 basal-type cancers from microarray study were CK5/6-positive by immunohistochemistry, 12 of them were EGFR-positive, and 6 of them were c-KIT-positive [5]. However, these authors regarded as a positive case even the weakest reaction. They also found that EGFR-positivity was correlated with basal-type gene expression and was related with worse survival; the same applied to CK5/6-positive tumors. This observation is encouraging but it is still questionable that EGFR-positive tumors should be named as "basal-type". Fulford et al. found a good correlation with clinical outcome when as the "basal-like" tumors were only regarded the cases with the presence of keratin 14 [22].

Summarizing, we have demonstrated a discordance between real-time RT-PCR and immunohistochemistry in assessing basal-type cytokeratin status. This observation gives another difficulty in establishing an easy and simple method of identification of tumors that have a basal-like signature in microarray analysis.

## Competing interests

The authors declare that they have no competing interests.

## Authors' contributions

EP and AB carried out the molecular genetic studies, PP performed the statistical analysis and cooperated within final version of the manuscript, RKo conceived, designed and coordinated the study, RKu provided clinical and immunohistochemical data. All authors read and approved the final manuscript.
